# Enteroscrotal Fistula: A Rare Complication of Incarcerated Inguinal Hernia 

**Published:** 2010-12-01

**Authors:** Muhammad Sharif, Lubna Ijaz, Shahid Iqbal, Afzal Sheikh

**Affiliations:** Department of Pediatric Surgery, The Children's Hospital and the Institute of Child Health Lahore, Pakistan

**Keywords:** Strangulated inguinal hernia, Enteroscrotal fistula, Neonate

## Abstract

Inguinal hernia is a frequent surgical condition encountered of pediatric age group. It may get incarcerated and at times strangulated requiring prompt intervention. However if lesion is not treated timely a host of complications may occur. Enteroscrotal fistula is one such rare complication that may follow such discourse. We report a case of 25-days old male neonate who presented with enteroscrotal fistula due to incarcerated right inguinal hernia. Patient was explored through abdomen and the involved part of ileum was resected and ileo-ileal anastomosis performed.

## INTRODUCTION

Inguinal hernia is a common pediatric surgical disease. In about 12% to 17% of cases it gets incarcerated [1,2]. Incarceration is comparatively common below the age of one year. Most of the cases of incarcerated inguinal hernia are managed by early reduction, under sedation, followed by elective herniotomy after 48 hours; however, in about 10% patients emergency operations have to be performed [1,3].

Enteroscrotal fistula due to incarceration of inguinal hernia is a rare entity. To date only eight cases have been reported in Pubmed [3]. We report a case of 25-day-old male neonate in whom an enteroscrotal fistula developed due to incarceration of right inguinal hernia. 

## CASE REPORT

A 25-day-old male neonate presented in surgical emergency with complaints of feculent discharge from the scrotum, abdominal distension, and vomiting for two days. There had been a history of inguinoscrotal swelling which was ignored by the parents. At presentation, his vital signs were normal except for pyrexia (101°F). On systemic examination, abdomen was distended with visible bowel loops. On local examination there was wound in right hemiscrotum with hyperemic margins and feculent material coming through the wound. He was diagnosed as a case of enteroscrotal fistula and a laparotomy was performed through right lower transverse incision. Operative findings were strangulated inguinal hernia causing perforation of ileum leading to enteroscrotal fistula. Resection and end to end ileo-ileal anastomosis was performed and hernia was repaired from within the abdomen with purse-string sutures. Postoperative course was uneventful.

**Figure:**
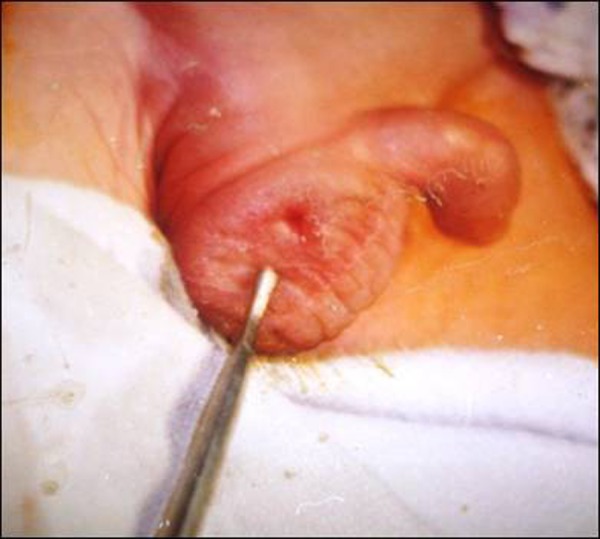
Figure 1: Showing opening of the enteroscrotal fistula.

## DISCUSSION

A delay in the treatment of inguinal hernia may lead to incarceration and strangulation. Various series have reported incidence of incarceration ranging from 12% to 31% [1,4]. However, the risk of strangulation following incarceration is very low (up to1.8%) [5]. Strangulated inguinal hernia may lead to enteroscrotal fistula formation [6].

In developing countries the risk of incarceration and hence complications is much higher due to delay in seeking treatment. This may be the result of lack of awareness among parents, general practitioners, and even pediatrician about the timings of surgery for inguinal hernia in neonates and children.

With spontaneous enteroscrotal fistula emergency is usually over and patient is managed electively after initial stabilization. Surgical approach is made either through inguinal region or abdomen. We performed laparotomy in our patient. To conclude, early diagnosis and management of inguinal hernia may prevent many sinister problems of delayed presentation.

## Footnotes

**Source of Support:** Nil

**Conflict of Interest:** None declared
